# The RASSF gene family members RASSF5, RASSF6 and RASSF7 show frequent DNA methylation in neuroblastoma

**DOI:** 10.1186/1476-4598-11-40

**Published:** 2012-06-13

**Authors:** Anna Djos, Tommy Martinsson, Per Kogner, Helena Carén

**Affiliations:** 1 Department of Clinical Genetics, Institute of Biomedicine, University of Gothenburg, Sahlgrenska University Hospital, SE-413 45, Gothenburg, Sweden; 2 Medical Genomics, UCL Cancer Institute, University College London, London, UK; 3 Childhood Cancer Research Unit, Department of Woman and Child Health, Karolinska Institute, Karolinska Hospital, SE-17176, Stockholm, Sweden

## Abstract

**Background:**

Hypermethylation of promotor CpG islands is a common mechanism that inactivates tumor suppressor genes in cancer. Genes belonging to the *RASSF* gene family have frequently been reported as epigenetically silenced by promotor methylation in human cancers. Two members of this gene family, *RASSF1A* and *RASSF5A* have been reported as methylated in neuroblastoma. Data from our previously performed genome-wide DNA methylation array analysis indicated that other members of the *RASSF* gene family are targeted by DNA methylation in neuroblastoma.

**Results:**

In the current study, we found that several of the *RASSF* family genes (*RASSF2*, *RASSF4*, *RASSF5*, *RASSF6*, *RASSF7*, and *RASSF10*) to various degrees were methylated in neuroblastoma cell lines and primary tumors. In addition, several of the *RASSF* family genes showed low or absent mRNA expression in neuroblastoma cell lines. *RASSF5* and *RASSF6* were to various degrees methylated in a large portion of neuroblastoma tumors and *RASSF7* was heavily methylated in most tumors. Further, CpG methylation sites in the CpG islands of some *RASSF* family members could be used to significantly discriminate between biological subgroups of neuroblastoma tumors. For example, *RASSF5* methylation highly correlated to *MYCN* amplification and INRG stage M. Furthermore, high methylation of *RASSF6* was correlated to unfavorable outcome, 1p deletion and *MYCN* amplification in our tumor material.

**In conclusion:**

This study shows that several genes belonging to the *RASSF* gene family are methylated in neuroblastoma. The genes *RASSF5*, *RASSF6* and *RASSF7* stand out as the most promising candidate genes for further investigations in neuroblastoma.

## Introduction

Neuroblastoma (NB) is the most commonly occurring solid extra-cranial tumor in children accounting for 6% of cancer incidence and 9% of cancer deaths in children [1]. It is a highly clinically and biologically heterogeneous cancer of the postganglionic sympathetic nervous system with tumors developing from immature or dedifferentiated neural crest cells [1,2]. Most tumors originate in the adrenal medulla or in paraspinal sympathetic ganglia. Common genetic alterations in NB tumors are *MYCN* amplification, 17q gain, 1p deletion and loss of 11q [2,3]. The list of genes epigenetically silenced in cancer is growing and the inactivated genes represent all cellular pathways. Several genes have been reported as silenced by methylation in NB and one example is the Ras-associated family member *RASSF1A*, located at chromosome 3p [4]. CpG island methylation of *RASSF1A* has been reported as a frequent event in NB tumors and cell lines [4] and loss of heterozygozity (LOH) at 3p, i.e. the loci containing the *RASSF1A* gene, has been reported in primary NB tumors [5]. *RASSF1A* is also epigenetically silenced by promoter methylation in many other human tumors [6]. The Ras proto-oncogenes belong to a super-family of GTPases that participate in a range of cellular processes such as cell growth, adhesion, migration, differentiation and apoptosis [7], with defects in Ras signaling pathway resulting in disease and oncogenesis. The Ras proteins carry out their diverse functions via interaction with RASS effectors which have conserved Ras interacting domains. One of many such Ras interacting domains is the RA-domain, and the RA-domain is a common feature of the genes in the Ras-association domain family (RASSF). This family has ten members; *RASSF1-10,* which are divided into two groups, the classical members *RASSF1-6* and the N-terminal members *RASSF7-10*[8]. The classical *RASSF* family members have been reported to be involved in many biological processes such as microtubule stability, cell cycle control and apoptosis and are generally considered as tumor suppressors [8]. Based on our previous data using IIumina 27K methylation arrays [9] we noted that several of the *RASSF* genes were methylated in NB. Eight of the *RASSF* genes were included on the IIumina 27K methylation arrays *(RASSF1A, RASSF2, RASSF3, RASSF4, RASSF5, RASSF6, RASSF7* and *RASSF8)*. The following seven RASSF genes were chosen for further methylation analysis; *RASSF2, RASSF4, RASSF5, RASSF6, RASSF7, RASSF8* and *RASSF10).* The two CpG sites in *RASSF3* were unmethylated in all NB tumors and this gene was therefore not investigated further. *RASSF8* however, we wanted to include in the verification analysis with BSP to see if surrounding CpG sites also were unmethylated since this gene has been reported as methylated in Childhood Leukemia cell lines [10]. *RASSF1A* was not analyzed further as this gene is well known to be deregulated in NB due to DNA methylation. Recent published data have shown that *RASSF10* is methylated in other cancers which led us to include this gene in our analyses. In addition to the *RASSF1A* gene, DNA methylation was found in six out of seven analyzed *RASSF* genes (*RASSF2, 4, 5–7* and *10*). Several of the *RASSF* genes had reduced mRNA expression levels in NB cell lines and the methylation status of some of the *RASSF* genes was able to significantly discriminate between biological subgroups of NB tumors.

## Material and methods

### Cell lines and tumor material

A panel of nine NB cell lines; Kelly, NB69, SK-N-SH, SH-SY-5Y, SK-N-AS, SK-N-BE(2), SK-N-DZ, SK-N-FI and IMR-32 were used for analysis of DNA methylation status. All nine NB cell lines were subjected to epigenetic drug treatment and expression analysis with end-point RT-PCR or qRT-PCR. In addition, we have previously generated cDNA microarray data for SK-N-AS, SK-N-BE(2), SK-N-DZ and IMR-32 [9]. Data from Illumina Human Methylation27K DNA analysis BeadChips from fifty-nine primary NB tumors (Table 1) were also used, together with four NB cell lines, SK-N-AS, SK-N-BE(2), SK-N-DZ and IMR-32, one adrenal sample, unmethylated and methylated controls (EpiTect control DNA, Qiagen, Hilden, Germany) [9]. Control for the genomic content and the authenticy of all cell lines have been performed and genomic profiles of the cell lines generated [11]. Furthermore, short tandem repeat fingerprinting/genotyping of all the cell lines used were performed to verify the identity of cell lines, as described earlier [11].

**Table 1:** Patient data

**Tumor id**	**Outcome**	**INRG**	**age at diagnosis**	**age at follow up**	**1p-del**	**MNA**	**11q-del**	**17q-gain**
10E6	NED	M	<18 m	>60 m	pos	pos	neg	pos
10E7	DOD	M	>18 m	<60 m	neg	neg	neg	pos
10R2	DOD	M	<18 m	<60 m	pos	pos	neg	pos
10R4	NED	MS	<18 m	>60 m	neg	neg	neg	neg
10R8	DOD	L	>18 m	<60 m	neg	neg	pos	neg
11E1	NED	M	>18 m	>60 m	neg	neg	pos	pos
11E8	NED	L	<18 m	>60 m	neg	neg	neg	neg
11R9	DOD	M	>18 m	<60 m	neg	neg	pos	pos
12E5	NED	MS	<18 m	>60 m	neg	neg	neg	neg
12R1	NED	M	<18 m	>60 m	pos	neg	neg	pos
12R6	DOD	M	>18 m	<60 m	pos	pos	neg	neg
12R9	NED	M	>18 m	>60 m	neg	pos	neg	neg
13R0	DOD	M	>18 m	<60 m	neg	pos	pos	pos?
13R1	DOD	L	>18 m	<60 m	neg	pos	pos	pos
15R8	NED	L	<18 m	>60 m	pos	neg	pos	pos
16E2	NED	L	<18 m	>60 m	neg	neg	neg	neg
16E9	DOD	M	>18 m	<60 m	neg	pos	neg	pos?
16R4	NED	L	>18 m	>60 m	neg	pos	neg	pos
17E2	NED	L	>18 m	>60 m	neg	neg	neg	neg
17E4	DOD	M	>18 m	<60 m	neg	pos	pos	pos
18E2	NED	L	>18 m	>60 m	pos	neg	neg	pos
18E4	DOD	M	<18 m	<60 m	neg	pos	pos	pos
18E5	NED	L	>18 m	>60 m	neg	neg	neg	neg
18E7	NED	L	>18 m	>60 m	neg	neg	neg	pos
18E8	NED	L	<18 m	>60 m	neg	neg	neg	neg
19R1	NED	L	>18 m	>60 m	neg	neg	neg	neg
19R6	DOD	L	<18 m	<60 m	neg	pos	pos	pos
20R8	NED	L	>18 m	>60 m	neg	pos	pos	pos
23R2	NED	L	>18 m	>60 m	neg	pos		
23R4	NED	L	<18 m	>60 m	neg	neg	neg	neg
25R6	DOD	M	>18 m	<60 m	neg	pos	pos	pos
25R7	NED	L	<18 m	>60 m	neg	neg	neg	neg
26R0	NED	M	>18 m	>60 m	pos	neg	pos	pos
26R1	NED	L	<18 m	>60 m	neg	neg	neg	neg
27R7	NED	L	<18 m	>60 m	neg	neg	neg	neg
28R2	NED		<18 m	>60 m	neg	pos	pos	pos
30R0	NED	M	>18 m	>60 m	pos	neg	neg	neg
32R2	NED	M	>18 m	>60 m	pos	neg	pos	pos
34R0	DOD	M	>18 m	<60 m	neg	neg	neg	pos
34R5	NED	L	<18 m	<60 m	neg	neg	neg	neg
35R5	NED	L	<18 m	<60 m	neg	neg	neg	neg
35R7	DOD	M	<18 m	<60 m	pos	neg	neg	neg
36R1	DOD	M	>18 m	<60 m	pos	neg	neg	pos
36R2	NED	M	>18 m	<60 m	pos	neg	neg	pos
36R3	DOD	MS	<18 m	<60 m	neg	neg	neg	pos
37R5	NED	L	<18 m	<60 m	neg	neg	neg	neg
37R6	NED	L	<18 m	<60 m	neg	neg	neg	neg
38R6	NED	L	>18 m	<60 m	neg	neg	pos	neg
39R1	NED	M	>18 m	<60 m	neg	pos	pos	pos
3E2	DOD	M	>18 m	<60 m	pos	neg	neg	pos
43R2	DOD	M	<18 m	<60 m	neg	neg	neg	pos
43R3	NED	M	>18 m	<60 m	neg	pos	pos	pos
4E1	DOD	M	>18 m	<60 m	pos	neg	neg	pos
5E1	NED	L	<18 m	>60 m	neg	neg	neg	neg
6E9	DOD	L	>18 m	<60 m	pos	neg	pos	pos
8E4	NED	L	<18 m	>60 m	neg	neg	neg	neg
8E7	NED	L	>18 m	>60 m	neg	neg	neg	neg
9E5	DOD	M	>18 m	<60 m	neg	pos	pos	pos
9R9	DOD	M	>18 m	<60 m	pos	neg	pos	pos

NED, no evidence of disease; DOD, dead of disease; L, localized; M, metastatic; m, months.

1p-del, 1p-deletion; MNA, MYCN amplification; 11q-del, 11q-deletion; neg, negative; pos, positive.

### Analysis of DNA methylation

1 μg of genomic DNA was bisulfite modified using the EpiTect kit (Qiagen) according to the manufacturer’s instructions. Methylation status of seven *RASSF* family genes was investigated using bisulfite sequencing, methylation-specific PCR (MSP) or combined bisulfite restriction analysis (COBRA).

#### Methylation-specific PCR (MSP) and bisulfite sequencing

PCR amplifications were performed according to CarÃ©n et al. [9]. Primers were designed with the Bisearch software [12] and are listed in Table 2. One fully methylated control sample, one unmethylated control sample and one 50/50 mixture of methylated and unmethylated control (EpiTect) were used to optimize the reaction conditions and to ensure that the bisulfite modified DNA samples were equally amplified despite their methylation status. PCR products were visualized on a 2% agarose gel with GelRed (Biotinum, Hayward, CA). The methylation status of *RASSF2A* was determined using MSP and the methylation status of *RASSF5*, *RASSF7*, *RASSF8* and *RASSF10* were analyzed with bisulfite sequencing [9].

**Table 2:** Primers used in this study

**Gene**	**Method**	**Primer sequence**	**Product (bp)**	**AT °C**	**Primer design**
*RASSF2A*	MSP	F: 5′-GTTCGTCGTCGTTTTTTAGGCG-3′	109	62	[13]
		R: 5′-AAAAACCAACGACCCCCGCG-3′			
*RASSF2A*	MSP	F: 5′-AGTTTGTTGTTGTTTTTTAGGTGG-3′	109	60	[13]
		R: 5′-AAAAAACCAACAACCCCCACA-3′			
*RASSF4*	COBRA	F: 5′-AGGATAYGATATATGTAGTGGTTTTTGGATT-3′	270	TD 65-55	[14]
		R: 5′-ATTATAACCCCTAAATTACTTAACAAAAATACCAAA-3′			
RASSF5A*	BSP	F: 5′-TTAGGAAAGAGGAATATTTTAT-3′	434	TD 60-50	[12]
		R: 5′-TAAACCTTCAACCCTACCTCTTTC-3′			
RASSF5C**	BSP	F: 5′-GGGGTTTAGAGTTAGGGGTTTA-3′	345	TD 60-50	[12]
		R: 5′-TATAACTTTATCCCTTTACTA-3′			
*RASSF6*	COBRA	F: 5′-GTATAGGGAGTGGTTTAGGTTTTTTGATAT-3′	353	TD 67-57	[10]
		R: 5′-ATCCCCATTTTTTACCTATTATTCACACTATA-3′			
*RASSF7*	BSP	F: 5′-GAGAAAAGTTAGGTTTTAGA-3′	592	TD 62-52	[12]
		R: 5′-CTCAACAACCTTCTAATATAA-3′			
*RASSF8*	BSP	F: 5′-TTTTATAATGTAGYGTTGGYGTTTTAGTTT-3′	374	TD 67-57	[10]
		R: 5′-CRAAACTCRACRAAACTAAACRAAAAACT-3′			
*RASSF10*	BSP	F: 5′-TTGTTTTTGTTGTTTTYGTYGTTTTAGTAGATT-3′	634	TD 67-57	[10]
		R: 5′-CRATTAAACTTAACCAATTTACRAAAAACCTTA-3′			
*RASSF2A*	qRT-PCR	F: 5′-AAGGGGTGGAGAGTGATATGAAGAG-3′	194	60	[15]
		R: 5′-AGGGACGTTTGGTGGCTGTAGT-3′			
*RASSF4*	qRT-PCR	F: 5′-GGACTGCGCGATGACTGGAC-3′	126	56	[15]
		R: 5′-CCGACTTCTGAATGGACTTGCTGT-3′			
*RASSF5*	End-point RT-PCR	F: 5′-CCTGGGCATGAAACTGAGTGAAGA-3′	188	56	Manual
		R: 5′-tgatggcatctaggggcaggtaga-3′			
*RASSF6*	End-point RT-PCR	F: 5′-ATGGAGAGACTGAAGATGGC-3′	203	56	[15]
		R: 5′-CAGGGTGTTGCTGTGATAAG-3′			
*RASSF7*	End-point RT-PCR	F: 5′-CAGCAGAGCGAGCCTTGCAGGCTCA-3′	149	59	Manual
		R: 5′-CTGAGTGCCAGGAGGGCCCCTGTCA-3′			
*RASSF10*	qRT-PCR	F: 5′-CCATGACCCAGGAGAAACAG-3′	226	60	[10]
		R: 5′-TGCTGGCGAATTGTGTGGTC-3′			

*cg17558126, **cg02589695.

MSP, Methylation specific PCR; BSP, Bisulfite sequencing PCR; COBRA, Combined bisulfite restriction PCR.

qRT-PCR, Quantitative real time PCR; AT, Annealing temperature; TD, Touch down.

#### Combined bisulfite restriction analysis (COBRA)

The methylation status of *RASSF4* and *RASSF6* was determined using COBRA [16]. Bisulfite modified DNA was amplified as described above (primers in Table 2). For *RASSF4*, 2 μl of PCR product was incubated with 2U BstUI enzyme (CGCG) and 1xNEBuffer4 (New England BioLabs, Ipswich, MA) for 2 hours at 60°C. For *RASSF6*, 2 μl of PCR product was incubated with 1U of FastDigest TaqI (TCGA) and 1X FastDigest Green Buffer (Fermentas, Germany) for 15 minutes at 65°C. Digestion patterns were visualized on a 2% agarose gel with GelRed (Biotinum).

### Epigenetic drug treatments and expression analysis

Changes in gene expression following treatment with the demethylating agent, 5-Aza-2′-deoxycytidine (5-Aza-dC; Sigma-Aldrich co, St Louis, MO) or/and the histone deacetylase inhibitor trichostatin A (TSA; Sigma-Aldrich) were analyzed with either end-point RT-PCR or qRT-PCR using Sybergreen (Applied Biosystems, Foster City, CA). RNA extraction and cDNA synthesis were done as previously described [9]. End-point RT-PCR was used for *RASSF5*, *RASSF6* and *RASSF7.* PCR reactions were denatured at 96°C for 10 minutes, followed by 35 cycles of 96°C for 30 seconds, annealing temperature (Table 2) for 30 seconds and 72°C for 30 seconds ending with a 7 minute extension step at 72°C. PCR products were taken at different time points (cycle 25, 30 and 35) to ensure the detection of the amplification product in the exponential phase. The housekeeping gene *GUSB* was included as an endogenous control. The end-point RT-PCR products were run on a 2% agarose gel and fragments were visualized with UV-light using GelRed (Biotinum). Quantitation of end-point RT-PCR was performed with ImageJ 1.45 software (NIH, Bethesta, MO) and normalization was done using *GUSB*. For the genes *RASSF2A*, *RASSF4* and *RASSF10*, expression analysis was performed with qRT-PCR using Sybergreen (Applied Biosystems) and the SDS software was used to extract Ct-values. Quantification was performed with the standard curve method [17]. *GUSB* was used for normalization.

### Statistical analysis

The methylation beta-values from the CpG sites where methylation of the *RASSF* genes were detected were extracted from the 27K methylation arrays [9] and the difference in beta-values (methylation frequency) between different biological subgroups of NB were compared with Student’s two-sided *t*-test. The tests included INRG stage, 5-year overall survival (5-OS), *MYCN* amplification status, 1p deletion, and 11q deletion. Correction for multiple testing was done with Bonferroni correction. The IIumina ID’s of the CpG sites on the 27K methylation array are listed in Table 3.

**Table 3:** **Descriptives of the CpG sites from the** 27K **methylation array**

			**Methylation frequency (beta-value)**	
**Gene**	**ILMNID**^**a**^	**Relation to CGI**	**Mean**	**Range**
*RASSF1*	cg00777121	CGI	0.69	0.13-0.95
	cg06063729	CGI	0.02	0-0.05
	cg06821120	CGI	0.12	0.02-0.64
	cg06980053	CGI	0.20	0.06-0.59
	cg08047457	CGI	0.75	0.05-0.99
	cg11035216	CGI	0.02	0-0.03
	cg15043975	CGI	0.06	0.04-0.11
	cg21554552	CGI	0.61	0.04-0.96
	cg26357744	CGI	0.01	0-0.04
*RASSF2*	cg16884569	CGI	0.01	0-0.03
	cg19614321	CGI shore	0.06	0.01-0.27
*RASSF3*	cg07915282	CGI	0.02	0-0.07
	cg12157010	CGI	0.01	0-0.02
*RASSF4*	cg13603099	CGI shore	0.01	0-0.02
	cg17324128	CGI	0.10	0.04-0.24
*RASSF5*	cg01860753	CGI	0.18	0.04-0.38
	cg02589695	CGI	0.23	0.06-0.51
	cg08617916	CGI	0.01	0-0.02
	cg10167296	CGI	0.04	0.02-0.1
	cg17558126	CGI	0.13	0.03-0.43
	cg19452316	CGI	0.04	0-0.08
	cg22857604	CGI	0.04	0-0.1
	cg23520347	CGI	0.05	0.01-0.09
	cg24450312	CGI	0.09	0.02-0.16
*RASSF6*	cg03996822	CGI shore	0.39	0-0.94
	cg08647446	CGI	0.12	0.05-0.54
*RASSF7*	cg14896003	CGI shore	0.96	0.89-1.0
*RASSF8*	cg07469792	CGI	0.01	0-0.03
	cg22946876	CGI	0.00	0-0.03

ILMNID, Illumina ID; CGI, CpG island.

^a^http://www.illumina.com/Documents/products/technotes/technote_cpg_loci_identification.pdf

## Results

### Methylation analysis with methylation array, bisulfite sequencing, MSP and COBRA

Bisulfite sequencing, MSP and COBRA assays were performed in order to verify that the methylated CpG sites present on the 27K methylation array were indeed methylated and to explore if surrounding CpG sites had the same methylation status. Six of the seven *RASSF* genes were found to have methylated CpG islands in at least one NB cell line. Our 27K methylation array data showed dense *RASSF1A* methylation of NB primary tumors and cell lines which were expected since *RASSF1A* is well-known to be deregulated by DNA methylation in NB.

The *RASSF2A* MSP results confirmed the 27K methylation array data in that the cell line SK-N-AS showed low level of methylation. *RASSF2A* DNA methylation was generally not found in primary NB tumors; only a few cases with low grade methylation were detected.

The *RASSF4* gene region that was analyzed with COBRA showed very low level of methylation in three cell lines; SK-N-AS, IMR-32 and Kelly. The 27K methylation array showed that *RASSF4* are generally not methylated in primary NB tumors even though some tumors had low level of methylation (methylation frequency below 25%).

**Figure 1:**
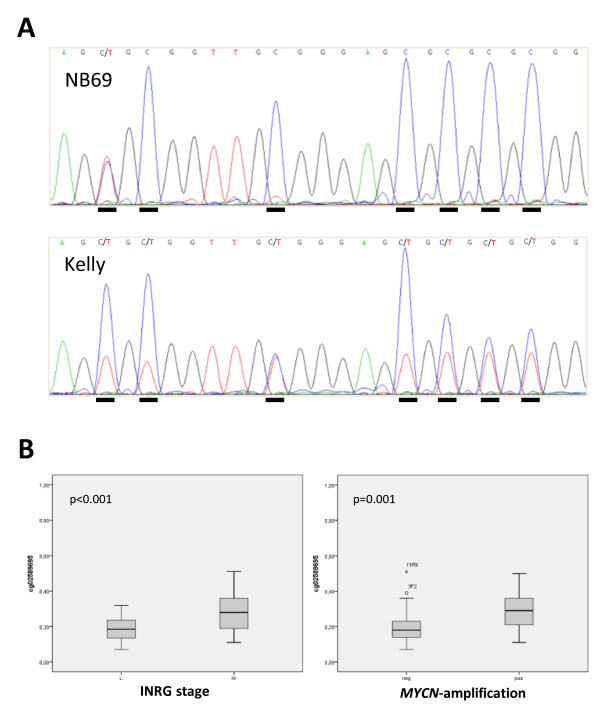
***RASSF5 *methylation and correlation to INRG stage and *MYCN *amplification. (A)** Examples of bisulfite sequencing of *RASSF5*in the *RASSF5A * CpG island: top sequence NB69 and bottom sequence Kelly. CpG sites are underlined and C in the sequence indicates methylation and C/T in the sequence indicate partial methylation. **(B)** High methylation of *RASSF5 * is significantly correlated to INRG stage M and *MYCN * amplification. P-values are indicated in the left upper corner in each graph. Box plot explanation; upper and lower hinges of the box represent the 75th percentile and 25th respectively; whiskers show highest and lowest values. Open circles represent outliers and asterisks show extremes.

Many of the primary NB tumors showed various levels of *RASSF5* methylation of at least one of the CpG sites present on the array, whereas some tumors were unmethylated at all sites present on the array (Table 3). SK-N-AS, IMR-32, SK-N-DZ and SK-N-BE(2) all showed partial methylation of *RASSF5* in at least one CpG site on the 27K methylation array. Two bisulfite sequencing assays were designed in order to confirm the *RASSF5* 27K methylation array results. One assay targeted the CpG rich region upstream of the promoter where the longest *RASSF5* transcript (*RASSF5A*) is initiated. The other targeted a CpG rich region in the promoter where the medium-sized *RASSF5* transcript *(RASSF5C)* is initiated. Bisulfite sequencing showed that the CpG site cg17558126, located just upstream of *RASSF5A* was methylated in 7/9 NB cell lines and the site cg02589695, located in *RASSF5C* was methylated in 6/9 NB cell lines. For example the NB cell line NB69 was fully methylated whereas Kelly was partially methylated at the CpG sites in the *RASSF5A* fragment (Figure 1A). Bisulfite sequencing of *RASSF5* thus confirmed the 27K methylation array results for both CpG sites. In NB primary tumors, the methylation frequency (methylation beta-values) of two of the CpG sites on the 27K methylation array were significantly higher in INRG stage M compared to stage L (p-values <0.001, Bonferroni corrected <0.02 for all sites), (Figure 1B). The methylation beta-values were also for two of the sites significantly higher in *MYCN*-amplified tumors compared to none-amplified (Bonferroni corrected p-values <0.02), (Figure 1B).

**Figure 2:**
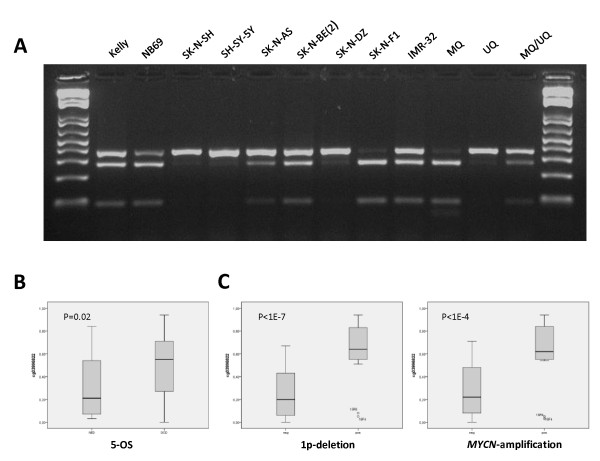
***RASSF6 *methylation and correlation to outcome, 1p deletion and *MYCN *amplification. (A)**Combined bisulfite restriction analysis of *RASSF6 *.PCR products are cleaved with TaqI. Samples from left to right: Kelly, NB69, SK-N-SH, SH-SY-5Y, SK-N-AS, SK-N-BE(2), SK-N-DZ, SK-N-FI, IMR-32, methylated control (MQ), unmethylated control (UQ) and a 50% mixture of methylated and unmethylated control (MQ/UQ). **(B)** High methylation of *RASSF6 * is correlated to an unfavorable outcome. **(C)** High methylation of *RASSF6 * is highly correlated to 1p deletion and *MYCN * amplification.

The 27K methylation array showed partial *RASSF6* methylation of at least one of the two CpG sites present on the array in all NB cell lines. The analyzed CpG island region of *RASSF6* was shown to be methylated in six out of nine (67%) cell lines according to our COBRA results (Figure 2A). The COBRA results thus confirmed partial methylation of *RASSF6* in IMR-32 and SK-N-BE(2) as well as the lower level of methylation in SK-N-AS. The methylation beta-values were significantly higher in patients with an unfavorable 5-year overall survival (Bonferroni corrected p-value <0.03), (Figure 2B). A higher methylation was also detected in 1p-deleted and *MYCN*-amplified tumors, with the most significant site located in the CpG island shore (Bonferroni corrected p-values of <5x10^-7^ and p < 2x10^-4^, respectively, Figure 2C).

**Figure 3:**
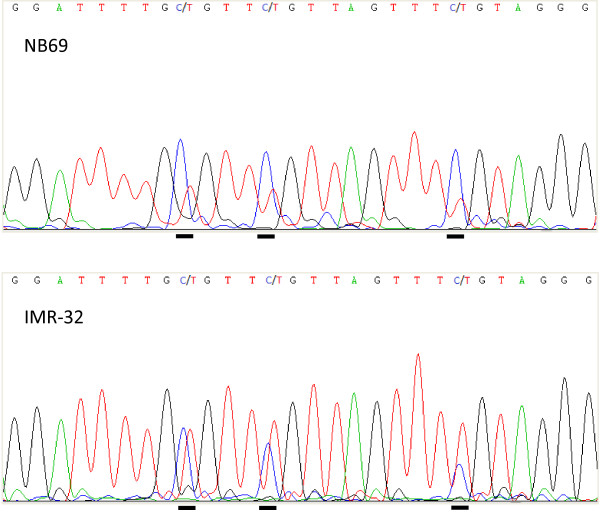
**Bisulfite sequencing of *RASSF7. ***Top sequence NB69 and bottom sequence IMR-32. Cytosines/thymines in the CpG dinucleotide are underlined. C in the sequence indicates methylation, C/T indicates partial methylation and T indicates unmethylated CpG sites.

The CpG site present on the 27K methylation array, located in the gene body of *RASSF7* was fully methylated in all four NB cell lines. Bisulfite sequencing of a fragment surrounding the transcriptional start site of *RASSF7* showed that eight of the nine (89%) NB cell lines were methylated, although to various levels (Figure 3). Bisulfite sequencing thus showed that the NB cell lines were methylated also around the *RASSF7* transcription start site.

*RASSF8* was unmethylated in all NB cell lines according to the 27K methylation array and bisulfite sequencing confirmed the array results. *RASSF8* was also unmethylated in the NB tumor material.

**Figure 4:**
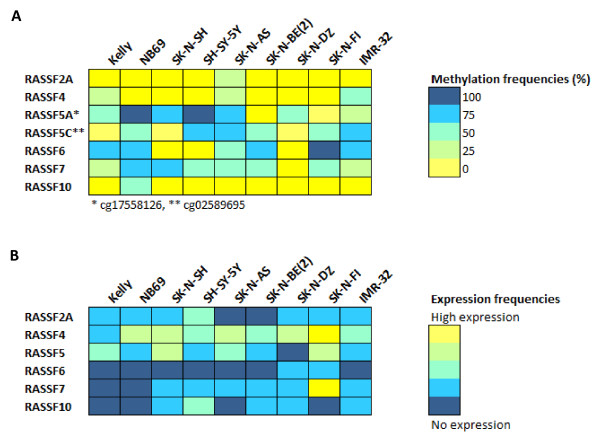
**Summary of *RASSF *gene expression and methylation. (A)** Summary of *RASSF * gene methylation in NB cell lines. Methylation frequencies for each gene and NB cell line are indicated with a color code where fully methylated is marked with blue and unmethylated is marked with yellow. **(B)** Summary of *RASSF * gene expression in NB cell lines. mRNA expression levels for each gene and NB cell line are marked with a color code where blue indicate absent expression and yellow high expression level.

*RASSF10* was unmethylated in eight of the nine (89%) cell lines whereas NB69 was partially methylated at all CpG sites. The methylation results are summarized in Figure 4A and the methylation frequencies of each CpG site on the 27K methylation array are listed in Table 3.

### Expression analysis and up-regulation after 5-aza-dC and TSA treatment

*RASSF2A* mRNA expression in NB cell lines was very low overall, with no difference in expression between methylated and unmethylated cell lines (Figure 4B). Quantitative real-time PCR showed that *RASSF2A* expression was up-regulated in 4/9 NB cell lines after 5-Aza-dC/TSA treatment (Table 4).

**Table 4:** Up-regulation of gene expression following treatment with 5-Aza-dC or 5-Aza-dC/TSA

		Rassf2A*	Rassf4*	Rassf5**	Rassf6**	Rassf7**	Rassf10*
Kelly	AZA				UD		
	AZA/TSA		↑		UD	↑	↑
NB69	AZA			↑	UD		
	AZA/TSA	↑		↑	UD		
SK-N-SH	AZA				UD		
	AZA/TSA				UD		
SH-SY-5Y	AZA			↑	UD	↑	
	AZA/TSA	↑		↑	UD		
SK-N-AS	AZA		↑	↑	UD		
	AZA/TSA		↑		UD		
SK-N-BE(2)	AZA		↑	↑	UD		
	AZA/TSA		↑	↑	UD	↑	
SK-N-DZ	AZA			↑		↑	↑
	AZA/TSA			↑		↑	
SK-N-FI	AZA		↑				
	AZA/TSA	↑	↑				
IMR-32	AZA		↑	↑	UD		
	AZA/TSA	↑	↑	↑	UD		

Arrows indicate a more than 2 fold up-regulation of gene expression following treatment.

UD, undetermined - gene transcripts not detected in the PCR amplification.

*qRT-PCR, **End-point RT-PCR.

*RASSF4* expression was present in all NB cell lines with a lower expression in Kelly, SH-SY-5Y, SK-N-BE(2) and IMR-32 (Figure 4B). The lower expression in Kelly, IMR-32 and SK-N-BE correlated with the presence of methylation according to either COBRA or 27K methylation array results. Further, the three cell lines (SK-N-AS, IMR-32 and SK-N-BE(2)), that showed low level of methylation on the 27K methylation array were up-regulated following treatment with 5-Aza-dC and even more up-regulated following a combined treatment with 5-Aza-dC and TSA. The methylated cell line Kelly also showed up-regulation of *RASSF4* following a combined treatment with 5-Aza-dC and TSA (Table 4).

**Figure 5:**
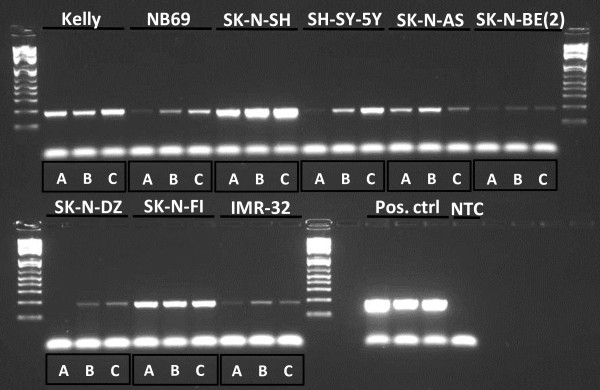
**Up-regulation of *RASSF5 *expression following treatment with 5-Aza-dC and TSA. ***RASSF5 *end-point RT-PCR samples from left to right: Kelly A, B, C, NB69 A, B, C, SK-N-SH A, B, C, SH-SY-5Y A, B, C, SK-N-AS A, B, C, SK-N-BE(2) A, B, C, SK-N-DZ A, B, C, SK-N-FI A, B, C, IMR-32 A, B, C. Three positive controls (Pos. ctrl) and one non template control (NTC). A = untreated, B = AZA treated and C = AZA/TSA treated.

*RASSF5* expression varied between the cell lines and *RASSF5* mRNAs was up-regulated after epigenetic treatment in 6/9 of the NB cell lines (see Figure 4B, Figure 5 and Table 4). The highest level of up-regulation was seen in the cell lines IMR-32 and SH-SY-5Y.

*RASSF6* was not expressed in seven out of the nine (78%) NB cell lines and the remaining two had expression just above the detection limit. No up-regulation was seen following treatment with either 5-Aza-dC alone or in conjunction with TSA (Figure 4B and Table 4).

*RASSF7* expression was overall very low in the NB cell lines with exception for SK-N-FI which had moderate expression (Figure 4B). *RASSF7* expression was strongly up-regulated in the methylated cell line SK-N-BE(2) after treatment with 5-Aza-dC in conjunction with TSA with both cDNA microarray and end-point RT-PCR. *RASSF7* was up-regulated in 4/9 NB cell lines following at least one of the treatments (Table 4).

Very low *RASSF10* expression was detected in five of the nine (56%) NB cell lines (Figure 4B). *RASSF10* expression was not up-regulated following epigenetic treatment in most of the NB cell lines (Table 4).

## Discussion

In this study, we investigated whether the *RASSF* family genes are epigenetically silenced in NB. Data from our previously performed 27K methylation array showed that members of the *RASSF* gene family were methylated in NB cell lines and tumors [9]. The methylation status observed with the 27K methylation array was verified by DNA methylation analysis using bisulfite sequencing, MSP or COBRA, of the corresponding CpG island. DNA methylation was most commonly observed in *RASSF7* (eight out of nine cell lines methylated), *RASSF5* (six to seven out of nine cell lines methylated depending on region analyzed) and *RASSF6* (six out of nine cell lines methylated) (Figure 4A). Gene expression analysis performed on NB cell lines showed in general low to moderate expression of the *RASSF* genes (Figure 4B). *RASSF4* had the highest expression in the NB cell lines whereas the mRNA levels of *RASSF6*, *RASSF7* and *RASSF10* were either below detection levels or very low in most NB cell lines. Also, the mRNA levels of *RASSF2A* and *RASSF5* were low in general. In order to see if *RASSF* gene expression could be restored, NB cell lines were treated with 5-Aza-dC/or TSA. Gene expression of most of the *RASSF* genes (*RASSF2A*, *RASSF4*, *RASSF5*, *RASSF7* and *RASSF10*) was up-regulated following epigenetic treatment, suggesting these genes may be epigenetically regulated. The concentration of each epigenetic drug and the treatment time will most certainly affect the results of up-regulation. The chosen conditions of drugs and treatment times were based upon optimization conditions where re-expression of a panel of known methylated genes occurred [9]. More gene-specific optimizations of the epigenetic drug treatment conditions may be necessary in order to explore the re-activation potential fully. Also, up-regulation of a gene may be the result of their up-stream regulators being affected by treatment and it is also possible that other epigenetic mechanisms than DNA methylation are responsible for the up-regulation. Genes belonging to the *RASSF* family are generally considered as TSGs and many of the members have been reported as silenced by promoter methylation in human cancers, (Table 5).

**Table 5:** **Information of the*RASSF*genes studied in relation to tumorigenesis**

**Symbol**	**Chr**	**Product**	**Methylated in cancer**	**Comment**
*RASSF1A*	3p21	Ras association domain family member 1	NB tumors and cell lines [4] as well as various human cancers, reviewed in [6]	TSG involved in regulation of cell proliferation. Promotes apoptosis and cell cycle arrest, involved in migration and maintenance of genomic stability (reviewed in [6]). KO of *RASSF1A* in mice enhances spontaneous tumor formation [18]; [19]
*RASSF2*	20p13	Ras association domain family member 2	Colorectal cancer [13], gastric cancer [20], nasopharyngeal carcinoma [21], breast-, lung and NSCLC tumors [22], thyroid cancer [23], pancreatic cancer [24]	Function as a TSG, reduces colony formation, promotes apoptosis and cell cycle arrest [25]; [15]; [21]; [22]; [23]
*RASSF4*	10q11	Ras association domain family member 4	Kidney-, breast- and lung cancer cell lines, breast- and lung primary tumors [14]	Have growth inhibitory properties and promotes apoptosis in lung- and breast tumor cell lines [14]
*RASSF5*	1q32	Ras association domain family member 5	NB cell lines [26]; [27], lung-, breast-, colorectal-, and kidney tumor cell lines and in primary NSCLC tumors [28], Wilms tumor [29], CCRCC [30], gastric cancer [20], colon cancer [13], squamous cell cancer of head and neck [31], hepatocellular carcinoma [32]	Also called NORE1 and forms heterodimers with RASSF1A [33]. Associate with microtubules and act growth inhibitory by a process involving p53. Promotes apoptosis when overexpressed or in the presence of activated Ras [34]. Neuroblastoma tumors, especially non *MYCN*-amplified, show suppressed *NORE1A* expression [27]
*RASSF6*	4q13	Ras association domain family member 6	Childhood leukemia [10]	Putative TSG in childhood leukemia [10]. Promotes apoptosis [35]. Downregulated at both mRNA and protein level in gastric cancer. Gastric cancer patients with *RASSF6*-negative tumors had worse outcome and higher recurrence rate than patients with *RASSF6*-positive tumors [36]
*RASSF7*	11p15	Ras association domain family (N-terminal) member 7		Centrosome associated protein necessary for spindle formation and completion of mitosis in the neural tube in Xenopus [8]. Required for completion of mitosis in human cells and KO results in mitotic arrest [37]
*RASSF8*	12p12	Ras association domain family (N-terminal) member 8	Childhood leukemia cell lines [10]	TSG candidate in lung cancer [38]. KO enhances anchorage independent growth in soft agar and promotes tumor formation in mice [39]
*RASSF10*	11p15	Ras association domain family (N-terminal) member 10	Childhood leukemia [10], thyroid cancer [40], primary glioblastomas and astrocytomas [41], malign melanoma [42]	Suggested as a regulator of mitosis. Over-expression decrease colony formation in soft agar [41]

Chr, chromosome; TSG, tumor suppressor gene; KO, knock out; NSCLS, non-small cell lung cancer; CCRCC, clear cell renal cell carcinoma; LOH, loss of heterozygosity.

***RASSF1A*** is a TSG involved in a range of cellular processes that are essential for normal cell growth control. Rassf1a is one of the most commonly inactivated proteins in cancer and inactivation by promotor hypermethylation is a common event in various human malignancies, including NB [4]. Demonstrating the validity of our 27K methylation array data, we detected dense *RASSF1A* methylation of NB primary tumors and cell lines, which is in agreement with published data.

***RASSF2A*** mRNA expression was in the current study generally low in NB cell lines and up-regulation of *RASSF2A* was seen following a combined treatment with 5-Aza-dC and TSA (4/9 NB cell lines) even though methylation at the *RASSF2A* CpG island was not commonly observed.

***RASSF4*** mRNA expression was detected in all NB cell lines and 5-Aza-dC and TSA treatment resulted in up-regulation of *RASSF4* mRNA levels in 5/9 NB cell lines. The strongest up-regulation was detected in the cell line IMR-32 which also showed the highest methylation level.

***RASSF5***, also called *NORE1* (Novel Ras Effector 1), is localized at 1q32.1 and has a 60% similarity to *RASSF1A,* the most commonly described methylated gene in cancer so far. The *RASSF5* gene encodes at least three different isoforms due to different promoter usage and alternative splicing. Two of the *RASSF5* isoforms, *RASSF5A* and *RASSF5C* are broadly expressed in most normal tissues. *RASSF5A* is the longest isoform transcribed from the most 5′-promoter and the isoform *RASSF5B* is produced by alternative splicing. The shorter isoform *RASSF5C* is transcribed from a more downstream promoter. Promoter methylation of *RASSF5A* has been reported to not occur in primary NB tumors and there are some conflicting data concerning methylation of NB cell lines, where NB cell lines have been described as low methylated or unmethylated in different studies [26,27]. Interestingly *RASSF5* was recently shown to be demethylated and up-regulated in the NB cell line SK-N-BE during ATRA-induced differentiation [43], suggesting that *RASSF5* could be aberrantly methylated in undifferentiated NB tumors cells but demethylated and re-expressed through differentiation. According to our 27K methylation array data, two CpG sites were methylated in NB primary tumors and cell lines. The methylated CpG sites were located in different *RASSF5* promotor CpG islands. The 27K methylation array site cg17558126 was located in the most 5′-promoter where transcription of *RASSF5A* starts and cg02589695 were located in a downstream promoter were the *RASSF5C* transcript starts. Bisulfite sequencing of the two regions revealed that both CpG sites present on the 27K methylation array were indeed methylated in most NB cell lines (Figure 4A). The methylation status of the CpG sites surrounding cg17558126 (*RASSF5A*) was highly variable throughout the CpG island, but most sites showed partial methylation, whereas the CpG sites surrounding cg02589695 (*RASSF5C*) were unmethylated in all NB cell lines. The variable methylation of CpG sites in this island might explain why there are conflicting published data regarding the methylation status of *RASSF5A* in NB cell lines. Gene expression of *RASSF5A* have also been described as low in NB cell lines, with the highest expression in SK-N-SH and absent expression in IMR-32 [26], which is in agreement with our data (Figure 4A and 4B). *RASSF5* mRNA expression was in this study up-regulated for several NB cell lines. For example, the methylated cell line SH-SY-5Y was up-regulated after 5-Aza-dC treatment and even more up-regulated following a combined treatment with both 5-Aza-dC and TSA (Table 4). Two of the analyzed *RASSF5* CpG sites on the 27K methylation array were significantly more methylated in INRG stage M tumors compared to L tumors (Figure 1B). Also, *RASSF5A* methylation was highly correlated to *MYCN* amplification (Figure 1B). *RASSF5A* mRNA expression have also been reported as frequently down-regulated in NB and pheochromocytoma primary tumors and lower *RASSF5A* expression was seen in NB tumors without *MYCN*-amplification compared to *MYCN*-amplified tumors [27]. The methylation beta-value for two of the *RASSF5* sites was significantly higher in *MYCN*-amplified tumors compared to non-amplified tumors which contradicts an earlier report that showed lower expression of this gene in non-*MYCN*-amplified tumors [27].

***RASSF6***, located at chromosome region 4q13.3 has recently been suggested as a TSG candidate in childhood leukemia and was found to be silenced by heavy methylation across the whole CpG island in leukemia cell lines [10]. In the current study, *RASSF6* promoter methylation was found in 6/9 NB cell lines and *RASSF6* expression was absent or just above detection level in the panel of NB cell lines (Figure 4A and 4B). High methylation of *RASSF6* was significantly correlated to unfavorable outcome (5-OS), 1p deletion and *MYCN* amplification in our patient cohort (Figure 2B and 2C). Recently, *RASSF6* was shown to be down-regulated at both mRNA and protein level in gastric cancer tumors and loss of *RASSF6* expression correlated with poor survival and increased tumor recurrence rate [36]. Functional studies have indicated that *RASSF6* is involved in promoting apoptosis [35].

***RASSF7***, also known as *HRC1* (HRAS1 cluster 1), is located at chromosome region 11p15.5 and lacks the conserved SARAH domain present in *RASSF1-6*. To our knowledge, there are yet no reports of epigenetic silencing of *RASSF7* in cancer but important functions have been reported (Table 5). In this study, bisulfite sequencing showed methylation of the *RASSF7* promotor CpG sites in 8/9 NB cell lines (Figure 4A). All four NB cell lines present on the 27K methylation array were heavily methylated (84-96%) at the analyzed CpG site. Interestingly, the mRNA expression of *RASSF7* was very low or absent in most NB cell lines (Figure 4B). According to our cDNA microarray analysis the methylated NB cell line SK-N-BE was strongly up-regulated following epigenetic treatment, which was verified with end-point RT-PCR (Table 4).

***RASSF10***, located at 11p15.2, has recently been reported as methylated and silenced in childhood leukemia [10], thyroid cancer [40] and in astrocytic glioma [41]. In this study the *RASSF10* mRNA expression was absent or just above the detection level in NB cell lines but low methylation was found in only 1/9 NB cell lines (Figure 4A and B).

Collectively, the *RASSF* family members have been demonstrated to have several tumor suppressive properties (Table 5). Although RASSF proteins lack catalytic activity, they are suggested to be non-enzymatic adaptors that are involved in growth and tumor suppression. The molecular mechanisms behind their growth suppressing properties are not yet elucidated but a number of reports show association with microtubules or centromeres indicating that the *RASSF* genes are important in microtubule dynamics and mitosis. In addition, the *RASSF* family genes participate in regulation of apoptosis and epigenetic silencing of *RASSF* genes may contribute to cancer by preventing RAS induced-apoptosis. In a normal cell, there is an important balance between signaling pathways that promote survival and those who promote apoptosis. If *RASSF* genes are silenced, the pro-apoptotic effects of RAS signaling may be lost which may favor the balance towards the pro-survival PI3 kinase pathway. Future studies regarding the exact function of the *RASSF* family genes and their interacting partners are essential to elucidate the role that epigenetic silencing of *RASSF* genes might play in NB and cancer in general.

Several genes from various cellular pathways have been reported as epigenetically silenced by DNA methylation in NB. For example, Caspase-8 (*CASP8*) located at 2q33 was one of the first genes to be reported as methylated in NB [44]. Aberrantly methylated genes could in the future be used in clinical patient stratification as biomarkers or as therapeutic targets. Our group and many others have shown that DNA methylation of single genes or a selected group of genes, are able to predict patient outcome, for a review see Decock et al., [45]. Epigenetic inactivation of *RASSF1A* has been reported as associated with high risk disease, age >1 year and poor survival for NB patients [46]. Further, *RASSF1A* hypermethylation in serum from patients with NB has been reported as a reliable prognostic predictor [47].

In summary, in addition to *RASSF1A* which is already known as frequently methylated in NB, this study highlights the RASSF gene family members *RASSF5*, *RASSF6* and *RASSF7* as promising candidates for further analysis in NB. These three genes are targeted by DNA methylation in NB primary tumors and cell lines and show low levels of mRNA expression in NB cell lines. Also, CpG site specific DNA methylation of *RASSF5* and *RASSF6* was able to significantly discriminate between different subgroups of NB.

## Competing interests

The authors declare that they have no competing interests.

## Authors’ contributions

AD carried out the experiments, analyzed the results and drafted the manuscript. HC planned and coordinated the study, performed experimental and statistical analysis and revised the manuscript. TM and PK provided clinical information. All authors read and approved the final manuscript.
